# Association of baseline glycaemic status with BMD change after 12 doses of romosozumab: a retrospective cohort study of 170 patients

**DOI:** 10.3389/fendo.2026.1941784

**Published:** 2026-10-14

**Authors:** Kimberly H. Tsoi, Xi Xiong, Xiaodong Liu, Chun-Ho Wong, Carol H. Y. Fong, Matrix M. H. Fung, Eunice K. H. Leung, Alan C. H. Lee, Tai-Pang Ip, Kathryn C. B. Tan, Chi-Ho Lee, Yu-Cho Woo, Manju Chandran, Elena Tsourdi, David T. W. Lui

**Affiliations:** 1 Department of Medicine, School of Clinical Medicine, Li Ka Shing Faculty of Medicine, The University of Hong Kong, Hong Kong, Hong Kong SAR, China; 2 Department of Medicine, Queen Mary Hospital, Hong Kong, Hong Kong SAR, China; 3 Department of Non-communicable Disease Epidemiology, Faculty of Epidemiology and Population Health, London School of Hygiene and Tropical Medicine, London, United Kingdom; 4 Department of Surgery, School of Clinical Medicine, Li Ka Shing Faculty of Medicine, The University of Hong Kong, Hong Kong, Hong Kong SAR, China; 5 Department of Medicine, Alice Ho Miu Ling Nethersole Hospital, Hong Kong, Hong Kong SAR, China; 6 Department of Medicine, Tung Wah Hospital, Hong Kong, Hong Kong SAR, China; 7 Osteoporosis and Bone Metabolism Unit, Department of Endocrinology, Singapore General Hospital, Singapore, Singapore; 8 Duke-NUS Medical School, Singapore, Singapore; 9 Asia Pacific Consortium on Osteoporosis (APCO), Hong Kong, Hong Kong SAR, China; 10 Division of Endocinology, Diabetes, and Bone Diseases, Department of Medicine III, Technische Universität Dresden Medical Center, Dresden, Germany; 11 Center for Healthy Aging, Technische Universität Dresden Medical Center, Dresden, Germany

**Keywords:** bone density, diabetes mellitus, osteoporosis, prediabetes, romosozumab

## Abstract

**Objective:**

We evaluated whether dysglycaemia is an independent factor associated with bone mineral density (BMD) gain during romosozumab treatment.

**Methods:**

We included all patients who initiated and received 12 doses of romosozumab in two Osteoporosis Centres from January 2019 to March 2025 and had dual-energy X-ray absorptiometry performed at baseline and upon completion of romosozumab. The cohort was divided into three groups. Diabetes was defined by physician diagnosis, glycated haemoglobin (HbA1c) ≥ 6.5%, or anti-diabetic medication use. Among those without known diabetes, HbA1c 5.7%–6.4% defined prediabetes and HbA1c ≤ 5.6% defined normoglycaemia. Percentage of BMD (%BMD) gain while on romosozumab was calculated. Multivariable linear regression analysis was used to identify independent factors associated with %BMD gain.

**Results:**

A total of 170 patients were included (age 71.6 ± 9.6 years; 167 women, 3 men; body mass index (BMI) 21.0 ± 3.6 kg/m^2^): 59.4% had normoglycaemia, 25.9% prediabetes, and 14.7% diabetes (all type 2). Of these, 64.1% had prior antiresorptive exposure. Baseline mean BMD T-scores (95% CI) were −3.0 (−3.2, −2.9) (lumbar spine, LS), −2.9 (−3.1, −2.8) (femoral neck, FN), and −2.6 (−2.7, −2.4) (total hip, TH). In total, 69.4% had prior fractures. Overall %BMD gain was 12.1% (95% CI 10.5%–13.7%) (LS), 3.6% (95% CI 2.4%–4.7%) (FN), and 3.4% (95% CI 2.5%–4.3%) (TH). Across the three glycaemic categories, %BMD gain did not differ (LS: p = 0.821; FN: p = 0.776; TH: p = 0.985). HbA1c did not correlate with %BMD gain. Baseline BMD T-score and prior antiresorptive treatment were the independent factors inversely associated with %BMD gain.

**Conclusion:**

This real-world study showed BMD gains of 12% at the LS and 3.5% at the hip with 12 doses of romosozumab. No statistically significant association was identified between baseline glycaemic category and one-year BMD change during romosozumab treatment. However, the relatively small diabetes subgroup limits the precision of this estimate.

## Summary

One year of romosozumab led to a 12% bone mineral density (BMD) gain over at the spine and 3.5% over at the femur in 170 patients with osteoporosis. No statistically significant association was identified between baseline glycaemic category and one-year BMD change during romosozumab treatment. Higher baseline BMD T-score and prior anti-resorptive therapy predicted smaller gains, highlighting the relevance of proper treatment sequencing.

## Introduction

Type 2 diabetes affects 10% of the global population. It is now well recognised that type 2 diabetes is associated with increased fracture risk, with low bone turnover being one of the main pathophysiological mechanisms (1, 2). Guidelines have now recognised bone fragility as an important comorbidity to address when managing patients with type 2 diabetes (3). Anti-osteoporosis treatments, namely osteoanabolic and antiresorptive agents, are the mainstay of treatment of osteoporosis in patients with type 2 diabetes at high fracture risk.

Bone fragility in type 2 diabetes is characterised by reduced bone turnover caused by osteocyte dysfunction (4). Osteocyte dysfunction leads to impaired mechanotransduction (5). The formation of advanced glycation end-products from hyperglycaemia contributes to the deterioration in bone quality. Cortical porosity is also a key feature contributing to impaired bone quality in type 2 diabetes (6). Osteoanabolic agents appear to be an attractive treatment strategy for osteoporosis in type 2 diabetes patients who are at high fracture risk (7). Nonetheless, there is a paucity of data evaluating the role of glycaemia in the efficacy of osteoanabolic agents, which may inform clinical practice. Romosozumab is a sclerostin inhibitor and produces the largest short-term bone mineral density (BMD) gains among currently available osteoporosis therapies (8). In turn, sclerostin has been shown in studies of rodents and humans—though not in all studies—to be elevated in type 2 diabetes and to correlate with impaired bone turnover (4). Sclerostin is a potent soluble antagonist of the bone-anabolic canonical Wnt–β-catenin signalling pathway (6). A *post-hoc* analysis of 165 patients from the pivotal ARCH trial with diabetes treated with romosozumab over 12 months reported a 12% increase in areal BMD at the lumbar spine (LS), exceeding the 5% increase seen in the alendronate treatment group (9). Results were consistent with those observed in the ARCH trial in general (10). Diabetes was not systematically assessed in the large randomised trials of romosozumab, so it remains to be determined whether romosozumab-induced skeletal benefits are independent of glycaemic status.

We conducted a retrospective cohort study of patients with osteoporosis treated with 12 doses of romosozumab to evaluate the association between dysglycaemia and BMD gain after 12 doses of romosozumab.

## Methods

### Study design

This is a retrospective cohort study.

### Participants

This study involved patients with osteoporosis who initiated romosozumab at the Osteoporosis Centres of Queen Mary Hospital and Tung Wah Hospital in Hong Kong between 1 January 2019 and 31 March 2025. Sample size was not calculated *a priori*, and all consecutive patients were included. This was a treatment-completer cohort: we only included patients who completed 12 doses of romosozumab and had dual-energy X-ray absorptiometry (DXA) performed both at baseline (pre-romosozumab) and upon completion of one year of romosozumab. Exclusion criteria did not differ across glycaemic status. Patients who did not have reassessment DXA or those who stopped treatment early (including those who experienced adverse events) were excluded.

In both Centres, romosozumab was initiated according to local osteoporosis guidelines (11). Patients with osteoporosis who are in the very-high-risk category (i.e., multiple fractures, recent major osteoporotic fracture within 24 months, BMD T-score ≤ −3.0, or incident fracture while on antiresorptive treatment) are offered osteoanabolic treatment, for which romosozumab and teriparatide are the two available options in Hong Kong. (Abaloparatide is not available). A history of myocardial infarction or ischaemic stroke is a contraindication to romosozumab in Hong Kong. All patients with osteoporosis were administered cholecalciferol supplementation to ensure 25-hydroxyvitamin D levels ≥75 nmol/L. Low pre-treatment 25-hydroxyvitamin D levels were corrected during romosozumab treatment. In addition, calcium supplementation was prescribed if necessary to ensure adequate daily calcium intake of 1,000–1,200 mg per day. Adherence to calcium and vitamin D was checked during each monthly injection and reinforced if it was suboptimal. Therefore, patients likely adhered to calcium and vitamin D supplementation during romosozumab treatment.

DXA scans were performed using the same machine for each individual. Hologic Horizon A was used at Queen Mary Hospital and GE Lunar Prodigy Advance was used at Tung Wah Hospital, with DXA performed according to standard acquisition and analysis techniques recommended by the International Society for Clinical Densitometry. The least significant changes are 0.032 g/cm^2^ for the spine and 0.027 g/cm^2^ for the hip for Hologic Horizon A at Queen Mary Hospital, while least significant changes have not been established at Tung Wah Hospital. The two machines have not been cross-calibrated.

### Definitions of covariates

The following demographic and anthropometric variables were retrieved from the electronic health records: age, sex, body mass index (BMI; weight divided by height^2^). Prior major osteoporotic fractures (of the hip, vertebrae, proximal humerus, and distal radius) were retrieved through detailed chart review. Vertebral fractures included both clinical and morphometric fractures. The imaging was systematically reviewed when available, and the fractures were confirmed to be due to low-energy trauma based on chart review. Multiple fractures were counted when considering the history of major osteoporotic fractures. Baseline 25-hydroxyvitamin D levels were retrieved. These were measured with liquid chromatography-tandem mass spectrometry (LC-MS/MS) and reported in nmol/L. Baseline glycated haemoglobin (HbA1c) assessment was routine in the two Osteoporosis Centres. HbA1c was therefore available in all patients before romosozumab treatment. We retrieved HbA1c during the year before the first dose of romosozumab. Factors affecting HbA1c reliability (including anaemia, haemoglobinopathy, chronic kidney disease, and recent transfusion) were reviewed for each included patient and were not found to be present. All patients were classified into one of three glycaemic categories: diabetes, prediabetes, and normoglycaemia. Diabetes was defined as an HbA1c ≥ 6.5%, a previous physician diagnosis of diabetes, or the use of antidiabetic medications. Among those without known diabetes, prediabetes was defined as HbA1c 5.7%–6.4%, while normoglycaemia was defined as HbA1c < 5.7%. None of the included patients had anti-diabetic medications initiated for non-diabetic indications. In those not classified as having diabetes based on a known physician diagnosis or use of anti-diabetic medications, glycaemic status was defined based on the single baseline HbA1c measurement. Fasting glucose was not routinely measured. All patients with diabetes had their diagnosis reviewed by endocrinologists and were deemed to have type 2 diabetes in accordance with international guidelines (12). Among the diabetes subgroup, further information was retrieved: diabetes duration, presence of diabetic complications, and the specific classes of anti-diabetic medications. The components of the Charlson comorbidity index were retrieved (13). In this study, we presented the modified Charlson comorbidity index, which excluded the diabetes components from the score, as diabetes was the variable of interest in this study. Prior use of teriparatide and antiresorptive agents (bisphosphonates, denosumab, and raloxifene) was reviewed. The duration of bisphosphonate/denosumab use and the transition period between romosozumab and bisphosphonate/denosumab were recorded. Romosozumab was only used in a sequential setting in this study: none of the patients received romosozumab concomitantly with another anti-osteoporosis treatment.

### Definition of outcomes

We calculated the percentage of BMD (%BMD) gain at the LS, femoral neck (FN), and total hip (TH) during romosozumab treatment as the difference between pre- and post-romosozumab BMD divided by pre-romosozumab BMD.

Additional outcomes of interest included incident fragility fractures occurring within the year of romosozumab, hypocalcaemia, osteonecrosis of the jaw, and atypical femoral fractures. Since we defined a treatment-completer cohort, no major adverse cardiovascular events were expected to be recorded, as their occurrence would have been a contraindication to romosozumab use.

### Statistical analyses

All analyses were conducted using SPSS version 29. Data were presented as mean ± standard deviation (SD), median (interquartile range [IQR]), or number (percentage), as appropriate. The 95% confidence intervals were presented for BMD changes. Between-group comparisons were performed using an independent-samples t-test or ANOVA for continuous variables and the chi-square test or Fisher’s exact test for categorical variables. Correlation analyses between two continuous variables were conducted using Spearman’s correlation. Characteristics were compared across patients in the three glycaemic categories (normoglycaemia, prediabetes, and diabetes). Bonferroni’s correction was applied in view of multiple testing. Furthermore, multivariable linear regression analyses were conducted to identify factors independently associated with %BMD gain while on romosozumab at the LS, FN, and TH. Age, sex, BMI, and glycaemic status were forced into the multivariable models due to their clinical relevance, while other covariates with univariate significance (p < 0.1) were also included. The models were further adjusted for treatment centre, accounting for the two DXA models which were not cross-calibrated. In all multivariable regression analyses, assumptions of linearity, homoscedasticity, normality, multicollinearity, and the presence of outliers were assessed using residual diagnostics, Q–Q plots, variance inflation factors, and influence statistics.

Several subgroup analyses were performed. First, we evaluated the association between glycaemic control (HbA1c) and %BMD gain while on romosozumab within the group without diabetes. Second, we evaluated the associations of HbA1c or complexity of the anti-diabetic regimen (not on anti-diabetic medications, on non-insulin medications, and insulin-treated) with %BMD gain while on romosozumab within the diabetes subgroup. Third, we studied the subgroup of patients who transitioned from bisphosphonates to romosozumab to assess the association between the duration of bisphosphonate use and %BMD gain while on romosozumab. Fourth, we studied the subgroup of patients who transitioned from denosumab to romosozumab to assess the association between the duration of denosumab use and %BMD gain. The time interval between the final denosumab dose and the initiation of romosozumab was considered in the analyses.

An exploratory analysis was conducted to study the association of prior teriparatide treatment with the %BMD gain while on romosozumab. We described the %BMD gain while on romosozumab among patients treated with teriparatide (without antiresorptive exposure) and treatment-naïve patients (those who never received antiresorptive treatment). These two groups were chosen to minimise confounding by prior antiresorptive exposure.

We conducted two sensitivity analyses: (i) using absolute change in BMD at the LS, FN, and TH as the outcome measures, and (ii) modelling follow-up BMD adjusted for baseline BMD using analysis of covariance because of the potential for mathematical coupling when analysing %BMD change.

## Results

**Figure 1** shows the study flow diagram. After applying the exclusion criteria, 170 patients with osteoporosis were included in this analysis: 109 from Queen Mary Hospital and 61 from Tung Wah Hospital (**Table 1**). The mean age was 71.6 ± 9.6 years. Of these, 167 were women and 3 were men. Mean BMI was 21.0 ± 3.6 kg/m^2^. This cohort was characterised by a high-fracture-risk profile: 69.4% had prior major osteoporotic fractures, 23.5% had recent major osteoporotic fractures, and 64.1% had prior antiresorptive treatment. There was no difference in the sex distribution (p = 0.312) and glycaemic status (p = 0.652) between those excluded and the 170 included patients, although those excluded were older (74.5 ± 10.1 years vs. 71.6 ± 9.6; p = 0.008).

**Figure 1:**
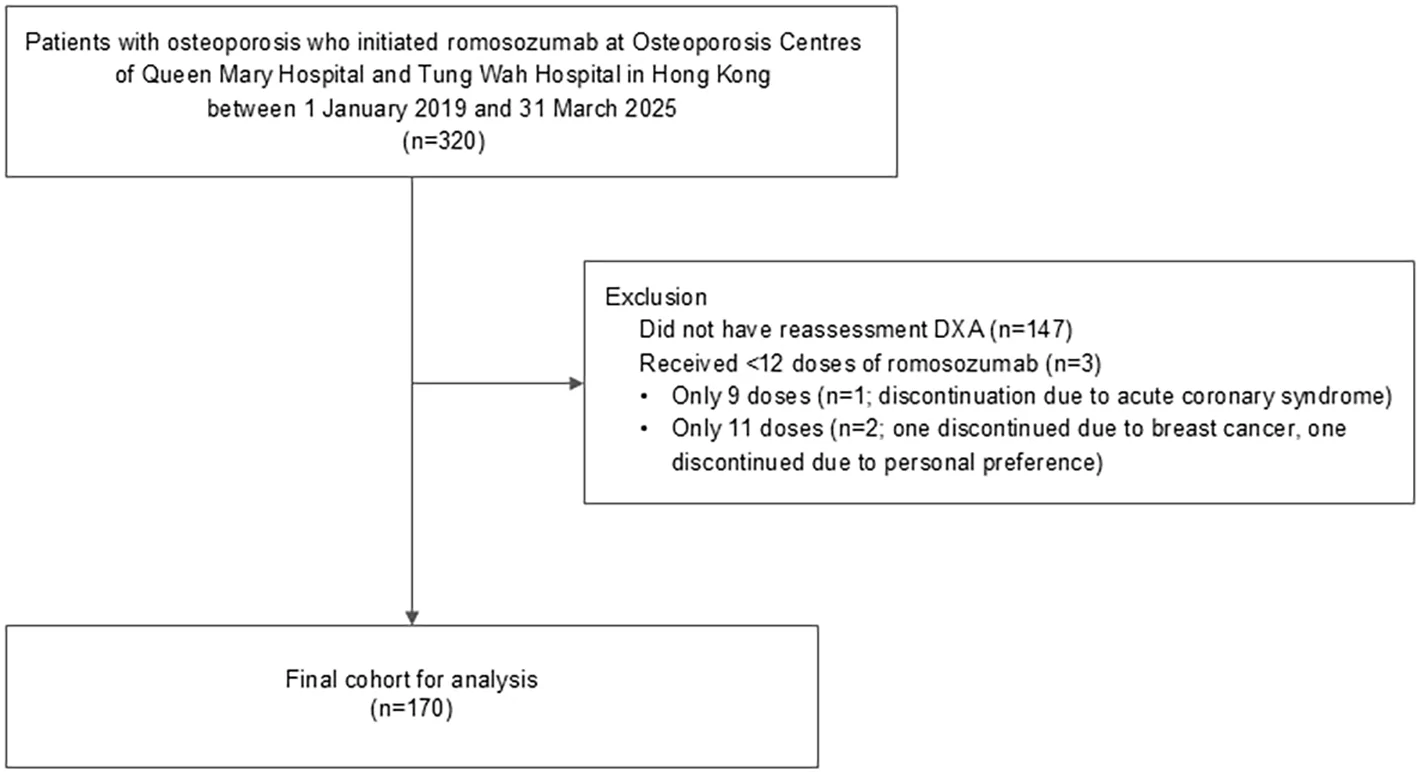
Study flow diagram.

**Table 1:** Characteristics of the cohort (n=170).

Characteristics	All	Queen Mary Hospital (n=109)	Tung Wah Hospital (n=61)	P-value
Age (years)	71.6 ± 9.6	72.4 ± 9.8	70.1 ± 9.1	0.119
Female	167 (98.2%)	108 (99.1%)	59 (96.7%)	0.293
BMI (kg/m^2^)	21.0 ± 3.6	20.9 ± 3.7	21.2 ± 3.5	0.581
eGFR (mL/min/1.73 m^2^)	87.0 ± 13.9	87.2 ± 14.2	86.6 ± 13.2	0.768
Prior teriparatide treatment	12 (7.1%)	10 (9.2%)	2 (3.3%)	0.215
Prior antiresorptive treatment	109 (64.1%)	83 (76.1%)	26 (42.6%)	**<0.001**
Duration of prior antiresorptives (years)	4.4 ± 3.7	5.1 ± 3.8	1.9 ± 1.8	**<0.001**
Prior bisphosphonate only	50 (29.4%)	42 (38.5%)	8 (13.1%)	**<0.001**
Prior denosumab only	26 (15.3%)	12 (11.0%)	14 (23.0%)	**0.034**
Prior raloxifene only	5 (2.9%)	4 (3.7%)	1 (1.6%)	-–
Prior bisphosphonate and denosumab	24 (14.1%)	21 (19.3%)	3 (4.9%)	**0.007**
Prior bisphosphonate and raloxifene	3 (1.8%)	3 (2.8%)	0 (0.0%)	-–
Prior denosumab and raloxifene	0 (0.0%)	0 (0.0%)	0 (0.0%)	-–
Prior bisphosphonate, denosumab and raloxifene	1 (0.6%)	1 (0.9%)	0 (0.0%)	-–
Prior major osteoporotic fractures	118 (69.4%)	77 (70.6%)	41 (67.2%)	0.383
Major osteoporotic fractures within 24 months before first dose of romosozumab	40 (23.5%)	26 (23.9%)	14 (23.0%)	0.526
Prior hip fracture	17 (10.0%)	16 (14.7%)	1 (1.6%)	**0.004**
Prior vertebral fracture	82 (48.2%)	54 (49.5%)	28 (45.9%)	0.384
Prior proximal humerus fracture	7 (4.1%)	6 (5.5%)	1 (1.6%)	0.213
Prior distal radius fracture	26 (15.3%)	19 (17.4%)	7 (11.5%)	0.210
Number of prior fractures	1 (0 – 2)	1 (0 – 2)	1 (0 – 1)	**0.025**
Modified Charlson Comorbidity Index	3 (2 – 3)	3 (2 – 3)	2 (2 – 3)	0.132
Prevalent cardiovascular diseases	0 (0.0%)	0 (0.0%)	0 (0.0%)	-–
25-hydroxyvitamin D (nmol/L)	85.5 ± 26.5	87.5 ± 27.4	81.8 ± 24.3	0.200
Serum albumin-adjusted calcium (mmol/L)	2.37 ± 0.10	2.36 ± 0.10	2.38 ± 0.11	0.134
Serum phosphate (mmol/L)	1.12 ± 0.17	1.11 ± 0.17	1.12 ± 0.16	0.748
Serum alkaline phosphatase (U/L)	78.2 ± 26.0	77.7 ± 27.8	78.9 ± 22.7	0.786
Baseline HbA1c (%)	5.7 ± 0.57%	5.7 ± 0.58%	5.8 ± 0.57%	0.481
Normoglycaemia	101 (59.4%)	71 (65.1%)	30 (49.2%)	0.253
Prediabetes	44 (25.9%)	21 (19.3%)	23 (37.7%)	
Diabetes	25 (14.7%)	17 (15.6%)	8 (13.1%)	
Baseline LS BMD (g/cm^2^)*	0.675 (0.655, 0.696) n=152	0.669 (0.652, 0.696) n=96	0.686 (0.654, 0.717) n=56	0.448
Baseline LS BMD T score*	-3.0 (-3.2, -2.9) n=152	-2.8 (-3.0, -2.6) n=96	-3.5 (-3.7, -3.2) n=56	**<0.001**
Baseline FN BMD (g/cm^2^)*	0.519 (0.501, 0.536) n=165	0.486 (0.467, 0.505) n=106	0.578 (0.548, 0.609) n=59	**<0.001**
Baseline FN BMD T score*	-2.9 (-3.1, -2.8) n=165	-3.0 (-3.2, -2.8) n=106	-2.8 (-3.1, -2.6) n=59	0.313
Baseline TH BMD (g/cm^2^)*	0.613 (0.597, 0.630) n=167	0.606 (0.585, 0.627) n=106	0.626 (0.598, 0.654) n=61	0.261
Baseline TH BMD T score*	-2.6 (-2.7, -2.4) n=167	-2.5 (-2.7, -2.3) n=106	-2.6 (-2.9, -2.4) n=61	0.426
% change in LS BMD*	12.1 (10.5, 13.7) n=142	10.5 (8.4, 12.5) n=92	15.1 (12.5, 17.7) n=50	**0.007**
% change in FN BMD*	3.55 (2.35, 4.74) n=153	2.60 (1.16, 4.04) n=97	5.20 (3.10, 7.29) n=56	**0.038**
% change in TH BMD*	3.41 (2.48, 4.34) n=160	2.56 (1.35, 3.77) n=100	4.83 (3.40, 6.26) n=60	**0.020**
Absolute change in LS BMD (g/cm^2^)*	0.078 (0.067, 0.089) n=142	0.065 (0.052, 0.078) n=92	0.101 (0.085, 0.118) n=50	**0.001**
Absolute change in FN BMD (g/cm^2^)*	0.017 (0.011, 0.023) n=153	0.011 (0.004, 0018) n=97	0.026 (0.016, 0.037) n=56	**0.013**
Absolute change in TH BMD (g/cm^2^)*	0.019 (0014, 0.025) n=160	0.014 (0.006, 0.021) n=100	0.029 (0.020, 0.037) n=60	**0.008**

*Data presented as mean (95% confidence interval).Bold values indicate statistical significance.

Baseline DXA was performed at a median of 2.7 months (IQR: 1.3–7.3) before the first dose of romosozumab: mean baseline BMD T-scores were −3.0 at the LS, −2.9 at the FN, and −2.6 at the TH (**Table 1**).

Eventually, 170 patients completed 12 doses of romosozumab. Reassessment DXA was performed at a median of 0.3 months after the last dose of romosozumab (IQR: from 0.8 months before to 1.4 months after). The median interval between baseline and follow-up DXA was 14.6 months (IQR: 12.8–20.5). Treatment with romosozumab resulted in BMD increases of 12.1% (95% CI 10.5%–13.7%) at the LS, 3.6% (95% CI 2.4%–4.7%) at the FN, and 3.4% (95% CI 2.5%–4.3%) at the TH. The respective absolute changes in BMD were +0.078 g/cm^2^ (95% CI + 0.067 to +0.089) at the LS, + 0.017 (95% CI + 0.011 to +0.023) at the FN, and +0.019 (95% CI + 0.014 to +0.025) at the TH. Among the subgroup of patients treated at Queen Mary Hospital with the least significant changes reported, 66/94 (70.2%) had an increase in LS BMD exceeding the least significant change, 31/105 (29.5%) had an increase in TH BMD exceeding the least significant change, 4/94 (4.3%) had a decrease in LS BMD exceeding the least significant change, and 12/105 (11.4%) had a decrease in TH BMD exceeding the least significant change.

In total, 6 patients sustained fragility fractures within one year of romosozumab treatment (4 in normoglycaemia, 2 in prediabetes, and 0 in diabetes; p = 0.581). None of the patients developed hypocalcaemia, osteonecrosis of the jaw, or atypical femoral fractures.

To evaluate the impact of dysglycaemia on the BMD gain while on romosozumab, we stratified the cohort into normoglycaemia (n = 101; 59.4%), prediabetes (n = 44; 25.9%), and diabetes (n = 25; 14.7%) (**Table 2**). Baseline BMD and T-scores did not differ across the three groups except for slightly higher FN BMD in prediabetes compared with normoglycaemia. The diabetes group was characterised by a smaller proportion of patients with prior antiresorptive treatment (normoglycaemia: 73.3%; prediabetes: 56.8%; diabetes: 40.0%; p = 0.004). %BMD gain was comparable across all three glycaemic categories at the LS (p = 0.821), FN (p = 0.776), and TH (p = 0.985). Results were similar when considering the absolute change in BMD across all three glycaemic categories. In the subgroup treated at Queen Mary Hospital with least significant changes determined, the proportion of patients who had an increase in BMD at the LS and TH exceeding the least significant change did not differ across glycaemic categories (LS: p = 0.704; TH: p = 0.643). Similarly, the proportion of patients who had a decrease in BMD at the LS and TH exceeding the least significant change did not differ across glycaemic categories (LS: p = 0.578; TH: p = 0.201). In the entire cohort of 170 patients, HbA1c did not correlate with %BMD gain at the LS (p = 0.660), FN (p = 0.363), and TH (p = 0.551).

**Table 2:** Comparison of characteristics across three glycaemic status (n=170).

Characteristics	Normoglycaemia (n=101)	Prediabetes (n=44)	Diabetes (n=25)	P value
Age (years)	72.3 ± 9.5	69.0 ± 9.1	73.1 ± 10.2	0.111
Female (%)	98 (97.0%)	44 (100%)	25 (100%)	0.352
Modified Charlson comorbidity index	3 (2 – 4)	2 (2 – 3)^a^	2 (1 – 3)^a^	**0.002**
BMI (kg/m^2^)	20.35 ± 3.39	22.25 ± 3.77^a^	21.22 ± 3.76	**0.013**
eGFR (mL/min/1.73 m^2^)	86.96 ± 13.31	88.21 ± 13.42	84.97 ± 16.83	0.649
Pretreated with teriparatide	6 (5.9%)	6 (13.6%)	0 (0%)	0.082
Pretreated with antiresorptive	74 (73.3%)	25 (56.8%)	10 (40.0%)^a^	**0.004**
Pretreated with bisphosphonates	53 (52.5%)	15 (34.1%)	10 (40.0%)	0.101
Pretreated with denosumab	34 (33.7%)	12 (27.3%)	3 (12.0%)	0.098
Major osteoporotic fractures within 24 months before first dose of romosozumab	24 (23.8%)	12 (27.3%)	4 (16.0%)	0.572
Prior nonvertebral fracture	39 (38.6%)	22 (50.0%)	12 (48.0%)	0.381
Prior vertebral fracture	53 (52.5%)	17 (38.6%)	12 (48.0%)	0.309
Prior hip fracture	14 (13.9%)	1 (2.27%)	2 (8.0%)	0.096
Prior proximal humerus fracture	3 (2.97%)	4 (9.10%)	0 (0.0%)	0.126
Prior distal radius fracture	15 (14.9%)	8 (18.2%)	3 (12.0%)	0.779
Number of prior fractures	1 (0 – 2)	1 (0 – 2)	1 (0 – 2)	0.807
25-hydroxyvitamin D (nmol/L)	84.49 ± 25.82	85.23 ± 27.84	90.43 ± 26.97	0.627
Albumin adjusted calcium (mmol/L)	2.36 ± 0.09	2.37 ± 0.11	2.41 ± 0.12	0.117
Serum phosphate (mmol/L)	1.11 ± 0.17	1.12 ± 0.18	1.13 ± 0.15	0.750
Serum alkaline phosphatase (U/L)	76.6 ± 26.9	79.5 ± 22.8	78.3 ± 28.7	0.831
Baseline HbA1c (%)	5.35 ± 0.27	5.89 ± 0.18^a^	6.62 ± 0.61^a,b^	**<0.001**
Baseline LS BMD (g/cm^2^) (n=152)^#^	0.666 (0.636, 0.695) n=90	0.702 (0.669, 0.735) n=39	0.668 (0.621, 0.714) n=23	0.322
Baseline LS BMD T score (n=152)^#^	-3.1 (-3.3, -2.8) n=90	-2.9 (-3.3, -2.6) n=39	-3.1 (-3.4, -2.7) n=23	0.851
Baseline FN BMD (g/cm^2^) (n=165)^#^	0.507 (0.483, 0.530) n=99	0.559 (0.530, 0.589)^a^ n=41	0.501 (0.450, 0.553) n=25	**0.030**
Baseline FN BMD T score (n=165)^#^	-3.0 (-3.1, -2.8) n=99	-2.7 (-3.0, -2.5) n=41	-3.1 (-3.5, -2.7) n=25	0.184
Baseline TH BMD (g/cm^2^) (n=167)^#^	0.610 (0.588, 0.632) n=100	0.639 (0.607, 0.670) n=42	0.586 (0.539, 0.632) n=25	0.133
Baseline TH BMD T score (n=167)^#^	-2.6 (-2.8, -2.4) n=100	-2.4 (-2.6, -2.1) n=42	-2.8 (-3.3, -2.4) n=25	0.136
% change in LS BMD (n=142)^#^	11.7 (9.63, 13.8) n=83	12.9 (9.57, 16.3) n=38	12.1 (7.39, 16.8) n=21	0.821
	12.8 (10.5, 15.0)*	12.9 (9.71, 16.0)*	12.2 (7.33, 17.1)*	0.977*
% change in FN BMD (n=153)^#^	3.72 (2.15, 5.29) n=91	3.75 (1.13, 6.37) n=39	2.52 (-0.23, 5.28) n=23	0.776
	3.77 (2.06, 5.49)*	3.41 (0.87, 5.86)*	5.27 (1.17, 9.37)*	0.761*
% change in TH BMD (n=160)^#^	3.47 (2.23, 4.72) n=94	3.36 (1.41, 5.32) n=41	3.25 (1.00, 5.51) n=25	0.985
	3.76 (2.47, 5.06)*	3.35 (1.47, 5.22)*	3.92 (1.10, 6.73)*	0.923*
Absolute change in LS BMD (g/cm^2^) (n=142)^#^	0.073 (0.059, 0.088) n=83	0.088 (0.066, 0.111) n=38	0.076 (0.048, 0.105) n=21	0.508
	0.082 (0.067, 0.096)*	0.086 (0.065, 0.107)*	0.080 (0.048, 0.112)*	0.929*
Absolute change in FN BND (g/cm^2^) (n=153)^#^	0.018 (0.011, 0.026) n=91	0.019 (0.005, 0.032) n=39	0.008 (-0.004, 0.021) n=23	0.506
	0.019 (0.011, 0.028)*	0.017 (0.005, 0.029)*	0.020 (0.0003, 0.040)*	0.939*
Absolute change in TH BMD (g/cm^2^) (n=160)^#^	0.020 (0.012, 0.027) n=94	0.020 (0.009, 0.032) n=41	0.017 (0.005, 0.028) n=25	0.914
	0.022 (0.014, 0.029)*	0.019 (0.008, 0.030)*	0.021 (0.005, 0.037)*	0.951*

LS, lumbar spine; FN, femoral neck; TH, total hip; BMD, bone mineral density.

^a^ p<0.05 with reference to normoglycaemia; ^b^p<0.05 with reference to prediabetes.

*adjusted for age, BMI, treatment centre, and modified Charlson Comorbidity Index using ANCOVA.

^#^ Data presented as mean (95% confidence interval).Bold values indicate statistical significance.

We evaluated the independent factors associated with %BMD gain at the LS, FN, and TH while on romosozumab, respectively. Baseline LS T-score (beta −2.75, 95% CI −4.29 to −1.21, p < 0.001) and prior antiresorptive treatment (beta −3.52, 95% CI −4.53 to −0.28, p = 0.033) were the independent negative factors associated with %BMD gain at the LS (**Table 3**). Baseline FN BMD T-score (beta −1.94, 95% CI −3.43 to −0.45, p = 0.011) and prior antiresorptive treatment (beta −2.55, 95% CI −5.05 to −0.05, p = 0.046) were the independent negative factors associated with %BMD gain at the FN. In addition, prior teriparatide treatment was an independent positive factor associated with %BMD gain at the FN (beta 5.87, 95% CI 0.34 to 11.4, p = 0.037) (**Table 4**). Baseline TH BMD T-score (beta −2.21, 95% CI −3.19 to −1.24, p < 0.001) and prior antiresorptive treatment (beta −2.65, 95% CI −4.55 to −0.75, p = 0.006) were the independent negative factors associated with %BMD gain at the TH. In addition, prior teriparatide treatment was an independent negative factor associated with %BMD gain at the TH (beta −4.43, 95% CI −7.97 to −0.89, p = 0.014) (**Table 5**). The interval between DXA assessments did not correlate with %BMD change at the LS, FN, and TH. Moreover, in all three multivariable linear regression models (**Tables 3**–**5**), the interaction term between glycaemic status and prior antiresorptive treatment was not significant in the models for %BMD change at the LS (p = 0.219), FN (p = 0.062), and TH (p = 0.659), respectively. Further adjustments for treatment centre (accounting for different DXA models) showed consistent results in all three regression models (**Supplementary Table 1–3**).

**Table 3:** Factors associated with percentage of BMD gain over lumbar spine (n=142).

Covariates	Univariate analysis		Multivariable analysis	
	Unadjusted beta (95% CI)	P-value	Adjusted beta (95% CI)	P-value
Female	-18.1 (-31.7, -4.58)	**0.009**	-12.8 (-24.9, -0.62)	**0.039**
Age (years)	-0.22 (-0.39, -0.04)	**0.016**	0.094 (-0.28, 0.47)	0.625
BMI (kg/m^2^)	-0.28 (-0.74, 0.19)	0.237	-0.12 (-0.55, 0.31)	0.588
Glycaemic status		0.821		0.480
Normoglycaemia	Reference	--	Reference	--
Prediabetes	1.22 (-2.62, 5.05)	0.531	1.42 (-2.03, 4.86)	0.420
Diabetes	0.36 (-4.42, 5.14)	0.882	-1.81 (-6.78, 3.16)	0.476
Modified CCI	-1.99 (-3.60, -0.38)	**0.016**	-1.78 (-5.27, 1.71)	0.318
Prior teriparatide	-6.40 (-12.7, -0.09)	**0.047**	-1.47 (-7.62, 4.69)	0.641
Prior antiresorptive	-6.27 (-9.42, -3.13)	<**0.001**	-3.52 (-4.53, -0.28)	**0.033**
Prior fracture	-3.65 (-7.01, -0.30)	**0.033**	-1.20 (-4.33, 1.92)	0.450
HbA1c (%)	-0.66 (-3.62, 2.30)	0.660	--	--
eGFR (mL/min/1.73 m^2^)	-0.02 (-0.14, 0.10)	0.715	--	--
Adjusted calcium (mmol/L)	4.27 (-12.7, 21.2)	0.618	--	--
Vitamin D (nmol/L)	-0.03 (-0.09, 0.04)	0.456	--	--
Baseline LS T-score	-3.75 (-5.22, -2.27)	**<0.001**	-2.75 (-4.29, -1.21)	**<0.001**

CCI, Charlson Comorbidity Index; HbA1c, Glycated hemoglobin; eGFR, estimated Glomerular Filtration Rate; Vitamin D, 25-hydroxyvitamin D.

The multivariable regression model included sex, age, BMI, and glycaemic status, in addition to variables with p <0.1 in the univariate analysis.

There was no significant association between the interval between DXA assessments and percentage of BMD gain over lumbar spine (p=0.867).Bold values indicate statistical significance.

**Table 4:** Factor associated with percentage of BMD gain over femoral neck (n=153).

Characteristics	Univariate analysis		Multivariable analysis	
	Unadjusted beta (95% CI)	P-value	Adjusted beta (95% CI)	P-value
Female	-7.29 (-15.9, 1.28)	0.095	-6.76 (-15.1, 1.58)	0.112
Age (years)	-0.05 (-0.18, 0.08)	0.450	-0.30 (-0.66, 0.06)	0.099
BMI (kg/m^2^)	-0.13 (-0.46, 0.19)	0.425	0.04 (-0.30, 0.37)	0.827
Glycaemic status		0.776		0.983
Normoglycaemia	Reference	--	Reference	--
Prediabetes	0.03 (-2.82, 2.87)	0.986	0.09 (-2.78, 2.96)	0.951
Diabetes	-1.20 (-4.67, 2.26)	0.494	0.43 (-4.17, 5.03)	0.854
Modified CCI	0.04 (-1.16, 1.24)	0.065	2.14 (-1.30, 5.58)	0.223
Prior teriparatide	5.87 (0.57, 11.2)	**0.030**	5.87 (0.34, 11.4)	**0.037**
Prior antiresorptive	-2.43 (-4.88, 0.03)	0.053	-2.55 (-5.05, -0.05)	**0.046**
Prior fracture	-0.36 (-2.92, 2.19)	0.779	--	--
HbA1c (%)	-0.99 (-3.12, 1.15)	0.363	--	--
eGFR (mL/min/1.73 m^2^)	0.05 (-0.03, 0.14)	0.213	--	--
Adjusted calcium (mmol/L)	-4.65 (-16.5, 7.18)	0.439	--	--
Vitamin D (nmol/L)	-0.02 (-0.07, 0.03)	0.343	--	--
Baseline FN T-score	-2.08 (-349, -0.67)	**0.004**	-1.94 (-3.43, -0.45)	**0.011**

CCI, Charlson Comorbidity Index; HbA1c, Glycated hemoglobin; eGFR, estimated Glomerular Filtration Rate; Vitamin D, 25-hydroxyvitamin D.

The multivariable regression model included sex, age, BMI, and glycaemic status, in addition to variables with p <0.1 in the univariate analysis.

There was no significant association between the interval between DXA assessments and percentage of BMD gain over femoral neck (p=0.661).Bold values indicate statistical significance.

**Table 5:** Factors associated with percentage of BMD gain over total hip (n=160).

Covariates	Univariate analysis		Multivariable analysis	
	Unadjusted beta (95% CI)	P-value	Adjusted beta (95% CI)	P-value
Female	1.74 (-5.16, 8.63)	0.619	4.60 (-1.51, 10.7)	0.140
Age (years)	-0.12 (-0.22, -0.02)	**0.017**	0.08 (-0.15, 0.32)	0.483
BMI (kg/m^2^)	-0.23 (-0.50, 0.04)	0.091	-0.07 (-0.35, 0.21)	0.629
Glycaemic status		0.985		0.265
Normoglycaemia	Reference	--	Reference	--
Prediabetes	-0.11 (-2.33, 2.11)	0.923	0.27 (-1.83, 2.36)	0.802
Diabetes	-0.22 (-2.89, 2.45)	0.870	-2.34 (-0.35, 0.21)	0.629
Modified CCI	-0.98 (-1.92, -004)	**0.042**	-1.21 (-3.37, 0.94)	0.269
Prior teriparatide	-3.86 (-7.51, -0.21)	**0.038**	-4.43 (-7.97, -0.89)	**0.014**
Prior antiresorptive	-3.29 (-5.13, -1.44)	**<0.001**	-2.65 (-4.55, -0.75)	**0.006**
Prior fracture	-2.53 (-4.50, -0.56)	**0.012**	-0.82 (-2.75, 1.11)	0.406
HbA1c (%)	-0.52 (-2.23, 1.19)	0.551	--	--
eGFR (mL/min/1.73 m^2^)	-0.0005 (-007, 0.07)	0.988	--	--
Adjusted calcium (mmol/L)	-2.58 (-11.6, 6.43)	0.573	--	--
Vitamin D (nmol/L)	-0.05 (-0.08, -0.01)	**0.015**	-0.03 (-0.06, 0.006)	0.105
Baseline TH T-score	-2.02 (-2.95, -1.08)	**<0.001**	-2.21 (-3.19, -1.24)	**<0.001**

CCI, Charlson Comorbidity Index; HbA1c, Glycated hemoglobin; eGFR, estimated Glomerular Filtration Rate; Vitamin D, 25-hydroxyvitamin D.

The multivariable regression model included sex, age, BMI, and glycaemic status, in addition to variables with p <0.1 in the univariate analysis.

There was no significant association between the interval between DXA assessments and percentage of BMD gain over total hip (p=0.211).Bold values indicate statistical significance.

### Subgroup analyses

Within the subgroup of patients without diabetes, baseline HbA1c did not correlate with %BMD gain at the LS (p = 0.554), FN (p = 0.962), and TH (p = 0.198).

We evaluated the subgroup of 25 patients classified as having diabetes. The median HbA1c was 6.6% (IQR: 6.4–6.9). The median duration of diabetes was 8 years (IQR: 2–23). Ten patients had at least one diabetic complication (3 had retinopathy and 8 had nephropathy; none had neuropathy or macrovascular complications). Regarding anti-diabetic medications, 19 were on metformin, 6 on sulfonylureas, 3 on thiazolidinediones, 4 on dipeptidyl peptidase-4 (DPP-4) inhibitors, 1 on a sodium–glucose cotransporter 2 (SGLT2) inhibitor, and 3 on insulin. None were on acarbose or glucagon-like peptide-1 (GLP-1) receptor agonists. Considering the complexity of anti-diabetic regimens, 3 patients were not on anti-diabetic medications, 20 were on non-insulin anti-diabetic medications, and 3 were insulin-treated. Baseline HbA1c did not correlate with %BMD gain at the LS (p = 0.660), FN (p = 0.363), and TH (p = 0.551). When categorising the diabetes subgroup according to the complexity of the anti-diabetic regimen, there was no significant difference in %BMD gain across the three groups (no medications, on non-insulin anti-diabetic medications, and insulin-treated) at the LS (p = 0.821), FN (p = 0.776), and TH (p = 0.985).

In all, 58 patients transitioned from bisphosphonates to romosozumab, and the median duration of bisphosphonate exposure was 4 years (IQR: 2.0–6.0). Romosozumab was initiated at a median of 1.5 months (IQR: 0–12) after the last dose of bisphosphonate. The transition interval between bisphosphonate and romosozumab did not correlate with %BMD change at the LS (p = 0.594), FN (p = 0.214), and TH (p = 0.272). The duration of bisphosphonate exposure did not correlate with %BMD gain at the LS (p = 0.610), FN (p = 0.593), or TH (p = 0.457).

A total of 46 patients transitioned from denosumab to romosozumab, and the median duration of denosumab exposure was 2.0 years (IQR: 1.0–3.5). In all, 36 patients (78.3%) transitioned from denosumab to romosozumab at 6 months after the last dose of denosumab. The duration of denosumab exposure (in years) inversely correlated with %BMD gain at the LS (beta −1.29, 95% CI −2.37 to −0.20, p = 0.022) and TH (beta −0.75, 95% CI −1.46 to −0.04, p = 0.038) and also showed a possible inverse correlation with %BMD gain at the FN, although the correlation did not reach statistical significance (beta −0.71, 95% CI −1.51 to −0.10, p = 0.083). The transition period between denosumab and romosozumab did not correlate with %BMD gain at the LS (p = 0.744), FN (p = 0.975), and TH (p = 0.803). Neither age nor BMI correlated with %BMD gain at the LS, FN, or TH.

### Exploratory analyses

To further explore the association between prior teriparatide exposure and %BMD response while on romosozumab, we described three patients who had been treated with teriparatide but not with antiresorptive agents, along with 60 patients who were treatment-naïve (**Supplementary Table 4**). Mean %BMD change at the LS was +5.8% (range: +0.3% to +11.3%) in the teriparatide-treated group and +15.1% (95% CI + 11.7% to +18.5%) in the treatment-naïve group. Mean %BMD change at the FN was −5.3% (range: −21.8% to +24.7%) in the teriparatide-treated group and +4.5% (95% CI + 2.2% to +6.8%) in the treatment-naïve group. Mean %BMD change at the TH was −1.7% (range: −11.8% to +11.4%) in the teriparatide-treated group and +5.9% (95% CI + 3.4% to +8.3%) in the treatment-naïve group.

### Sensitivity analyses

In the first set of sensitivity analyses using absolute changes in BMD as the outcome measures, the results were consistent with the primary analyses using %BMD change during romosozumab treatment (**Supplementary Table 5–7**). In the second set of sensitivity analyses, which modelled follow-up BMD adjusted for baseline BMD using analysis of covariance, the results were also consistent with the primary analyses using %BMD change during romosozumab treatment (**Supplementary Table 8**).

## Discussion

In this real-world study, 12 doses of romosozumab led to an average BMD gain of 12% at the LS and 3.5% at the femur. To our knowledge, this is the first report of the association between glycaemic status and romosozumab-induced BMD gain, with no statistically significant association identified. Such information fills an important evidence gap regarding the management of bone fragility in type 2 diabetes. In line with existing evidence, prior exposure to antiresorptive therapy attenuates BMD gains achieved with romosozumab, highlighting the crucial role of optimal treatment sequencing to attain maximal BMD benefits from anti-osteoporosis treatment.

Diabetes was seldom reported as a comorbidity in the existing real-world studies of romosozumab treatment. Only a recent study in the United Arab Emirates of 87 patients with osteoporosis reported lower BMD gain with romosozumab in the diabetes subgroup (n = 34), defined by a documented diagnosis and use of glucose-lowering medication (10). Notably, the diabetes group was older (70.2 ± 8.4 vs. 64.5 ± 14.9 years, p = 0.045) and had a higher total hip BMD T-score (−2.08 ± 1.52 vs. −2.29 ± 1.22, p = 0.064) in that study, which may have confounded the results. Furthermore, none of the existing studies has systematically measured HbA1c to identify diabetes. Our study has the strength of complete baseline HbA1c measurement, showing no association of glycaemic status or pre-treatment HbA1c with BMD gain. Within the diabetes subgroup, we did not identify any association between glycaemic control or anti-diabetic regimen complexity and %BMD gain. Nevertheless, our study only included 25 patients with diabetes, which may represent a heterogeneous group comprising newly diagnosed diabetes, long-standing diabetes, insulin-treated disease, and well-controlled disease. Multicentre studies are needed to shed light on how diabetes-specific risk factors of bone fragility may influence BMD response to romosozumab. For instance, duration of diabetes is one major determinant of skeletal fragility in diabetes. With longer disease duration, all the diabetes-specific risk factors for skeletal fragility would accumulate over time as additional ‘hits’ to an already-compromised skeleton (2). In turn, duration of diabetes may influence the response to romosozumab treatment.

Our study reported BMD gain with romosozumab consistent with existing real-world cohort studies (14–20), in which reported BMD gain ranged from 6.7%–15.4% at the LS, 2.3%–12.4% at the FN, and 2.9%–8.6% at the TH. The pivotal FRAME study reported a 13.3% BMD gain at the LS, 5.9% at the FN, and 6.9% at the TH (21), whereas the other pivotal ARCH trial reported a 13.7% increase in LS BMD and a 6.2% increase in TH BMD (10). It is important to note that when romosozumab was administered for 1 year after alendronate (STRUCTURE (22)) or denosumab (Phase 2 extension (23)), the BMD gain was attenuated (LS BMD increased by 9.8% and 5.3%, respectively, and TH BMD increased by 2.9% and 0.9%, respectively). In line with these, we showed that prior antiresorptive treatment was a significant negative factor associated with BMD response while on romosozumab. This may be explained by suppressed bone turnover and a reduction in the pool of osteoblast precursors available for activation due to prior antiresorptive treatment, and by persistently altered bone microarchitecture after treatment discontinuation (10, 14, 16, 20–24). A prior study suggested that longer prior exposure to antiresorptive treatment was associated with lower BMD gain on subsequent romosozumab therapy (20). Recent prospective studies in postmenopausal women have further shown that the residual effects of both denosumab and zoledronic acid may vary according to baseline C-terminal telopeptide of type I collagen (β-CTX) and BMI, with some individuals showing prolonged suppression beyond the conventional dosing interval (25, 26). These findings suggest that the type of previous antiresorptive agent, duration of treatment, timing of the last dose, baseline bone turnover status, and BMI may influence the subsequent BMD response to romosozumab. Indeed, our study and others have shown that the use of romosozumab following a long duration of denosumab treatment specifically warrants caution because of reports of a paradoxical drop in BMD when patients transitioned to romosozumab (20). Evaluations of the residual effects according to bone turnover status may offer additional insights. In addition, baseline BMD T-score has been shown to be a significant negative predictor of BMD response while on romosozumab (15–18). Taken together, the results reiterated the importance of proper treatment sequencing by initiating treatment with osteoanabolic agents, especially for those with a very low BMD T-score and those with very high fracture risk, as stated in various guidelines (11, 27).

Limited real-world evidence suggests that teriparatide may blunt the BMD gain while on romosozumab (28, 29), although the results were not consistent across studies and the attenuation tended to be less significant compared with prior antiresorptive treatment (30). In our limited dataset with only 12 patients with prior teriparatide exposure, we observed inconsistent associations between prior teriparatide exposure and %BMD change while on romosozumab (ranging from an inverse association at the TH to a neutral association at the LS and a positive association at the FN). In the exploratory analysis, %BMD change in the teriparatide-treated group appeared numerically lower than that in the treatment-naïve group. However, as there were only three teriparatide-treated patients included, the observation was exploratory and highly uncertain. Further investigations are warranted in this regard.

Our study has the strength of a systematic evaluation of glycaemic status in the entire cohort to enable evaluation of the association between dysglycaemia and BMD gain while on romosozumab. Nonetheless, our results should be interpreted taking into consideration several limitations. First, this was a retrospective study in which residual confounding could not be entirely excluded. Second, there may be treatment-completer selection bias and survivorship bias. Third, the cohort size was relatively small, especially the diabetes subgroup, limiting the statistical power. Type II error must be acknowledged. Fourth, among those not classified as having diabetes based on a known physician diagnosis or use of anti-diabetic medications, we only used a single baseline HbA1c measurement to classify their glycaemic status. We acknowledge the possibility of a transient elevation in HbA1c such that stable prediabetes might not be reliably distinguished from temporary dysglycaemia. Fifth, we did not have bone turnover markers or a trabecular bone score to provide insight into bone microarchitecture or bone turnover status. Sixth, our study was not powered to evaluate fracture outcomes. Seventh, with only three men in the cohort, we were unable to evaluate the sex-specific effect of romosozumab on BMD change. Eighth, this study included two centres that utilised two different DXA platforms, which required adjustment in statistical modelling to account for potential confounding. Ninth, as the cohort comprises predominantly Chinese patients, results should be extrapolated to other ethnicities with caution. Finally, longitudinal glycaemic data were not available to examine the association between changes in glycaemic status and BMD change while on romosozumab.

## Conclusion

In this retrospective treatment-completer cohort, one year of romosozumab was associated with mean BMD increases of approximately 12% at the LS and 3.5% at the hip. No statistically significant association was identified between baseline glycaemic category and BMD change. However, the small number of participants with type 2 diabetes and the observational design limit conclusions regarding equivalence of treatment response. Previous antiresorptive exposure, particularly prior denosumab treatment, may influence subsequent BMD response and warrants further study in larger, prospectively characterised cohorts.

## Data Availability

The raw data supporting the conclusions of this article will be made available by the authors, upon reasonable request.
