# Supplementary material for: Association of baseline glycaemic status with BMD change after 12 doses of romosozumab: a retrospective cohort study of 170 patients
Source: Front Endocrinol (Lausanne). 2026 Oct 14;17:1941784. doi: 10.3389/fendo.2026.1941784 (PMC13713262; doi:10.3389/fendo.2026.1941784)
Supplement: Supplementary file 1 [file DataSheet1.docx]

**Supplementary Table 1**	Factors associated with percentage of BMD gain over lumbar spine after adjustment for treatment centre (n=142)

|  | Adjusted beta  (95% CI) | p-value |
| --- | --- | --- |
| Female | -12.9 (-25.0, -0.71) | **0.038** |
| Age (years) | 0.10 (-0.28, 0.47) | 0.622 |
| BMI (kg/m^2^) | -0.12 (-0.55, 0.31) | 0.578 |
| Glycaemic status |  | 0.574 |
| Normoglycaemia | Referent | -- |
| Prediabetes | 1.20 (-2.30, 4.69) | 0.502 |
| Diabetes | -1.69 (-6.66, 3.29) | 0.507 |
| Modified CCI | -1.75 (-5.24, 1.73) | 0.324 |
| Prior teriparatide | -1.28 (-7.44, 4.89) | 0.685 |
| Prior antiresorptive | -3.26 (-6.57, 0.05) | 0.054 |
| Prior fracture | -1.26 (-4.38, 1.86) | 0.430 |
| Baseline LS T-score | -2.63 (-4.20, -1.05) | **0.001** |
| Center: QMH (Hologic) | -1.21 (-4.45, 2.03) | 0.465 |

**Supplementary Table 2.**	Factors associated with percentage of BMD gain over femoral neck after adjustment for treatment centre (n=153)

|  | Adjusted beta  (95% CI) | p-value |
| --- | --- | --- |
| Female | -6.15 (-14.5, 2.16) | 0.147 |
| Age (years) | -0.29 (-0.64, 0.06) | 0.106 |
| BMI (kg/m^2^) | 0.04 (-0.29, 0.37) | 0.814 |
| Glycaemic status |  | 0.991 |
| Normoglycaemia | Referent | -- |
| Prediabetes | -0.48 (-3.39, 2.43) | 0.746 |
| Diabetes | 0.51 (-4.06, 5.07) | 0.828 |
| Modified CCI | 2.07 (-1.35, 5.48) | 0.235 |
| Prior teriparatide | 6.01 (0.52, 11.5) | **0.032** |
| Prior antiresorptive | -1.85 (-4.45, 0.75) | 0.153 |
| Baseline FN T-score | -2.08 (-3.57, -0.59) | **0.006** |
| Center: QMH (Hologic) | -2.34 (-4.92, 0.23) | 0.074 |

**Supplementary Table 3**	Factors associated with percentage of BMD gain over total hip after adjustment for treatment centre (n=160)

|  | Adjusted beta  (95% CI) | p-value |  |
| --- | --- | --- | --- |
| Female | 4.84 (-1.27, 10.9) | 0.121 |  |
| Age (years) | 0.08 (-0.15, 0.31) | 0.495 |  |
| BMI (kg/m^2^) | -0.07 (-0.35, 0.21) | 0.629 |  |
| Glycaemic status |  | 0.357 |  |
| Normoglycaemia | Reference | -- |  |
| Prediabetes | -0.01 (-2.17, 2.14) | 0.990 |  |
| Diabetes | -2.19 (-5.22, 0.85) | 0.158 |  |
| Modified CCI | -1.19 (-3.33, 0.96) | 0.279 |  |
| Prior teriparatide | -4.10 (-7.68, -0.53) | **0.025** |  |
| Prior antiresorptive | -2.35 (-4.33, -0.38) | **0.020** |  |
| Prior fracture | -0.93 (-2.87, 1.01) | 0.348 |  |
| Vitamin D | -0.03 (-0.06, 0.006) | 0.102 |  |
| Baseline TH T-score | -2.14 (-3.12, -1.16) | **<0.001** |  |
| Center: QMH (Hologic) | -1.03 (-2.96, 0.91) | 0.299 |  |

**Supplementary Table 4**. Characteristics of patients treated with teriparatide and treatment-naïve patients

|  | Treatment-naïve patients (n=61) | Teriparatide patients (n=3) Mean (min – max) |
| --- | --- | --- |
| Age (years) | 68.4 ± 8.9 | 76 (74 – 87) |
| Female (%) | 59 (96.7%) | 3 (100%) |
| Modified Charlson comorbidity index | 2 (2 – 3) | 3 (3 – 4) |
| BMI (kg/m^2^) | 21.24 ± 3.89 | 25.49 (21.91 – 26.59) |
| eGFR (mL/min/1.73 m^2^) | 88.13 ± 11.16 | 94.92 (92.53 – 98.20) |
| Prior fracture (%) | 31 (50.8%) | 3 (100%) |
| 25-hydroxyvitamin D (nmol/L) | 77.23 ± 21.71 | 59 (37 – 157) |
| Albumin adjusted calcium (mmol/L) | 2.38 ± 0.10 | 2.35 (2.35 – 2.48) |
| HbA1c at baseline (%) | 5.83 ± 0.68 | 5.8 (5.7 – 6.1) |
| Baseline LS BMD (g/cm^2^) | 0.668 ± 0.105 | 0.696 (0.599 – 0.773) |
| Baseline LS BMD T score | -3.29 ± 0.79 | -2.80 (-4.1 - -2.0) |
| Baseline FN BMD (g/cm^2^) | 0.549 ± 0.116 | 0.581 (0.449 – 0.624) |
| Baseline FN BMD T score | -2.86 ± 0.87 | -2.60 (-3.5 - -2.1) |
| Baseline TH BMD (g/cm^2^) | 0.621 ± 0.099 | 0.630 (0.527 – 0.641) |
| Baseline TH BMD T score | -2.58 ± 0.86 | -2.70 (-3.3 - -1.8) |

**Supplementary Table 5.**	Factors associated with absolute change of BMD gain over lumbar spine (n=142)

|  | Univariate analysis | |  | Multivariable analysis | |
| --- | --- | --- | --- | --- | --- |
|  | Unadjusted beta  (95% CI) | p-value |  | Adjusted beta  (95% CI) | p-value |
| Female | -0.10 (-0.19, -0.01) | **0.030** |  | -0.07 (-0.16, 0.11) | 0.097 |
| Age (years) | -0.002 (-0.003, -0.0004) | **0.009** |  | -0.00004 (-0.003, 0.003) | 0.977 |
| BMI (kg/m^2^) | -0.0002 (-0.003, 0.003) | 0.862 |  | 0.0002 (-0.003, 0.003) | 0.918 |
| Glycaemic status |  | 0.784 |  |  | 0.484 |
| Normoglycaemia | Referent | -- |  | Referent | -- |
| Prediabetes | 0.003 (-0.02, 0.03) | 0.845 |  | 0.01 (-0.01, 0.04) | 0.341 |
| Diabetes | 0.009 (-0.02, 0.04) | 0.502 |  | -0.01 (-0.04, 0.03) | 0.620 |
| Modified CCI | -0.01 (-0.02, -0.003) | **0.011** |  | -0.009 (-0.03, 0.02) | 0.463 |
| Prior teriparatide | -0.03 (-0.07, 0.011) | 0.145 |  | -- | -- |
| Prior antiresorptive | -0.04 (-0.06, -0.02) | **<0.001** |  | -0.02 (-0.05, -0.002) | **0.031** |
| Prior fracture | -0.02 (-0.04, 0.004) | 0.100 |  | -- | -- |
| HbA1c (%) | -0.002 (-0.02, 0.02) | 0.801 |  | -- | -- |
| eGFR (mL/min/1.73 m^2^) | -0.0001 (-0.001, 0.001) | 0.830 |  | -- | -- |
| Adjusted calcium (mmol/L) | 0.014 (-0.10, 0.13) | 0.810 |  | -- | -- |
| Vitamin D (nmol/L) | -0.0002 (-0.0006, 0.0003) | 0.409 |  | -- | -- |
| Baseline LS T-score | -0.02 (-0.03, -0.005) | **0.004** |  | -0.01 (-0.02, 0.0004) | 0.060 |

CI, Confidence Interval; CCI, Charlson Comorbidity Index; HbA1c, Glycated hemoglobin; eGFR, estimated Glomerular Filtration Rate; Vitamin D, 25-hydroxyvitamin D.

In the multivariable regression analysis, the model included sex, age, BMI, and glycaemic status. Only variables with p < 0.1 in the univariate analysis were considered for inclusion

**Supplementary Table 6**	Factors associated with absolute change of BMD gain over femoral neck (n=153)

|  | Univariate analysis | |  | Multivariable analysis | | |
| --- | --- | --- | --- | --- | --- | --- |
|  | Unadjusted beta  (95% CI) | p-value |  | Adjusted beta  (95% CI) | p-value |  |
| Female | -0.03 (-0.08, 0.01) | 0.119 |  | -0.03 (-0.07, 0.02) | 0.237 |  |
| Age (years) | -0.0003 (-0.001, 0.0003) | 0.314 |  | -0.0002 (-0.001, 0.0004) | 0.512 |  |
| BMI (kg/m^2^) | -0.001 (-0.002, 0.001) | 0.425 |  | -0.00007 (-0.002, 0.002) | 0.936 |  |
| Glycaemic status |  | 0.506 |  |  | 0.290 |  |
| Normoglycaemia | Reference | -- |  | Reference | -- |  |
| Prediabetes | 0.0005 (-0.01, 0.01) | 0.946 |  | 0.002 (-0.01, 0.02) | 0.163 |  |
| Diabetes | -0.01 (-0.27, 0.01) | 0.266 |  | -0.01 (-0.03, 0.005) | 0.936 |  |
| Modified CCI | -0.0004 (-0.01, 0.01) | 0.896 |  | -- | -- |  |
| Prior teriparatide | 0.02 (-0.01, 0.05) | 0.175 |  | -- | -- |  |
| Prior antiresorptive | -0.01 (-0.03, -0.001) | **0.031** |  | -0.01 (-0.03, -0.001) | **0.037** |  |
| Prior fracture | -0.003 (-0.02, 0.01) | 0.620 |  | -- | -- |  |
| HbA1c (%) | -0.01 (-0.02, 0.003) | 0.151 |  | -- | -- |  |
| eGFR (mL/min/1.73 m^2^) | 0.0004 (-0.0001, 0.001) | 0.087 |  | 0.0003 (-0.00008, 0.001) | 0.120 |  |
| Adjusted calcium (mmol/L) | -0.02 (-0.08, 0.04) | 0.462 |  | -- | -- |  |
| Vitamin D (nmol/L) | -0.0001 (-0.0003, 0.0001) | 0.380 |  | -- | -- |  |
| Baseline FN T-score | -0.01 (-0.02, -0.001) | **0.033** |  | -0.01 (-0.02, -0.002) | **0.009** |  |

CI, Confidence Interval; CCI, Charlson Comorbidity Index; HbA1c, Glycated hemoglobin; eGFR, estimated Glomerular Filtration Rate; Vitamin D, 25-hydroxyvitamin D.

In the multivariable regression analysis, the model included sex, age, BMI, and glycaemic status. Only variables with p < 0.1 in the univariate analysis were considered for inclusion

**Supplementary Table 7**	Factors associated with absolute change of BMD gain over total hip (n=160)

|  | Univariate analysis | |  | Multivariable analysis | | |
| --- | --- | --- | --- | --- | --- | --- |
|  | Unadjusted beta  (95% CI) | p-value |  | Adjusted beta  (95% CI) | p-value |  |
| Female | 0.007 (-0.03, 0.05) | 0.734 |  | 0.02 (-0.01, 0.06) | 0.224 |  |
| Age (years) | -0.001 (-0.001, -0.0003) | **0.004** |  | 0.0003 (-0.001, 0.002) | 0.676 |  |
| BMI (kg/m^2^) | -0.001 (-0.003, 0.0004) | 0.138 |  | -0.0005 (-0.002, 0.001) | 0.588 |  |
| Glycaemic status |  | 0.914 |  |  | 0.255 |  |
| Normoglycaemia | Reference | -- |  | Reference | -- |  |
| Prediabetes | 0.001 (-0.01, 0.01) | 0.924 |  | 0.002 (-0.01, 0.02) | 0.734 |  |
| Diabetes | -0.003 (-0.02, 0.01) | 0.707 |  | -0.01 (-0.03, 0.004) | 0.132 |  |
| Modified CCI | -0.007 (-0.01, -0.001) | **0.017** |  | -0.01 (-0.02, 0.01) | 0.357 |  |
| Prior teriparatide | -0.03 (-0.05, -0.004) | **0.022** |  | -0.03 (-0.05, -0.01) | **0.016** |  |
| Prior antiresorptive | -0.02 (-0.03, -0.01) | **<0.001** |  | -0.02 (-0.03, -0.01) | **0.002** |  |
| Prior fracture | -0.02 (-0.03, -0.004) | **0.010** |  | -0.01 (-0.02, 0.01) | 0.448 |  |
| HbA1c (%) | -0.004 (-0.01, 0.006) | 0396 |  | -- | -- |  |
| eGFR (mL/min/1.73 m^2^) | 0.0001 (-0.0003, 0.0004) | 0.781 |  | -- | -- |  |
| Adjusted calcium (mmol/L) | -0.01 (-0.07, 0.04) | 0.687 |  | -- | -- |  |
| Vitamin D (nmol/L) | -0.0003 (-0.0005, -0.00004) | **0.021** |  | -0.0001 (-0.0003, -0.004) | 0.183 |  |
| Baseline TH T-score | -0.009 (-0.02, -0.003) | **0.002** |  | -0.01 (-0.02, -0.004) | **<0.001** |  |

CI, Confidence Interval; CCI, Charlson Comorbidity Index; HbA1c, Glycated hemoglobin; eGFR, estimated Glomerular Filtration Rate; Vitamin D, 25-hydroxyvitamin D.

In the multivariable regression analysis, the model included sex, age, BMI, and glycaemic status. Only variables with p < 0.1 in the univariate analysis were considered for inclusion

**Supplementary Table 8.** Analysis of Covariance for Independent Factors of BMD at Lumbar Spine, Femoral Neck, and Total Hip at Reassessment

|  | Model 1: BMD at LS | |  | Model 2: BMD at FN | |  | Model 3: BMD at TH | |
| --- | --- | --- | --- | --- | --- | --- | --- | --- |
|  | Estimated marginal mean difference between group  (95% CI) | Between Subjects Effects, p-value |  | Estimated marginal mean difference between group  (95% CI) | Between Subjects Effects, p-value |  | Estimated marginal mean difference between group  (95% CI) | Between Subjects Effects, p-value |
| Female vs Male | -0.076 (-0.161, 0.010) | 0.082 |  | -0.026 (-0.068, 0.016) | 0.226 |  | 0.022 (-0.014, 0.059) | 0.232 |
| Age (years) | N/A | 0.980 |  | N/A | 0.157 |  | N/A | 0.833 |
| BMI (kg/m^2^) | N/A | 0.981 |  | N/A | 0.838 |  | N/A | 0.602 |
| Glycaemic status |  | 0.376 |  |  | 0.953 |  |  | 0.280 |
| Normoglycaemia | Reference | -- |  | Reference | -- |  | Reference | -- |
| Prediabetes | 0.014 (-0.015, 0.044) | 0.247 |  | 0.001 (-0.017, 0.019) | 0.885 |  | 0.003 (-0.013, 0.018) | 0.703 |
| Diabetes | -0.009 (-0.052, 0.033) | 0.605 |  | -0.003 (-0.031, 0.025) | 0.809 |  | -0.013 (-0.035, 0.009) | 0.153 |
| Modified CCI | N/A | 0.492 |  | N/A | 0.281 |  | N/A | 0.442 |
| Prior teriparatide | -0.011 (-0.053, 0.032) | 0.629 |  | 0.020 (-0.007, 0.048) | 0.152 |  | -0.027 (-0.048, -0.006) | **0.013** |
| Prior antiresorptive | -0.027 (-0.049, -0.005) | **0.017** |  | -0.016 (-0.029, -0.003) | **0.012** |  | -0.019 (-0.030, -0.007) | **0.001** |
| Prior Fracture | -0.006 (-0.028, 0.016) | 0.590 |  | -- | -- |  | -0.005 (-0.016, 0.007) | 0.443 |
| Vitamin D | -- | **--** |  | -- | -- |  |  | 0.218 |
| Baseline BMD at LS | N/A | **<0.001** |  | -- | -- |  | -- | -- |
| Baseline BMD at FN | -- | **--** |  | N/A | **<0.001** |  | -- | -- |
| Baseline BMD at TH | -- | -- |  | -- | -- |  | N/A | **<0.001** |

LS, lumbar spine; FN, femoral neck; TH, total hip.
